# Efficient and Stable Delivery of Multiple Genes to Fish Cells by a Modified Recombinant Baculovirus System

**DOI:** 10.3390/ijms19123767

**Published:** 2018-11-27

**Authors:** Qian Wang, Jian Fang, Qihua Pan, Yizhou Wang, Ting Xue, Lingyu Li, Tiansheng Chen

**Affiliations:** 1 Key Laboratory of Freshwater Animal Breeding, Ministry of Agriculture, College of Fisheries, Huazhong Agricultural University, Wuhan 430070, China; qian.wang@webmail.hzau.edu.cn (Q.W.); 15527598323@163.com (J.F.); panqihua@webmail.hzau.edu.cn (Q.P.); 18627944916@163.com (Y.W.); xue2011ting124@126.com (T.X.); lilingyu@webmail.hzau.edu.cn (L.L.); 2 Collaborative Innovation Center for Efficient and Health Production of Fisheries in Hunan Province, Changde 41500, China; 3 Hubei Engineering Technology Research Center for Aquatic Animal Diseases Control and Prevention, Wuhan 430070, China

**Keywords:** recombinant baculovirus, fast titer determination, fish cells, stable gene delivery, WSSV ie1 promoter

## Abstract

The recombinant baculovirus has been widely used as an efficient tool to mediate gene delivery into mammalian cells but has barely been used in fish cells. In the present study, we constructed a recombinant baculovirus containing the dual-promoter cytomegalovirus (CMV) and white spot syndrome virus (WSSV) immediate-early gene 1 (ie1) (WSSV ie1), followed by a puromycin–green fluorescent protein (Puro-GFP, *pf*) or puromycin–red fluorescent protein (Puro-RFP, *pr*) cassette, which simultaneously allowed for easy observation, rapid titer determination, drug selection, and exogenous gene expression. This recombinant baculovirus was successfully transduced into fish cells, including *Mylopharyngodon piceus* bladder (MPB), fin (MPF), and kidney (MPK); *Oryzias latipes* spermatogonia (SG3); and *Danio rerio* embryonic fibroblast (ZF4) cells. Stable transgenic cell lines were generated after drug selection, which was further verified by Western blot. A cell monoclonal formation assay proved the stable heredity of transgenic MPB cells. In addition, a recombinant baculovirus containing a *pr* cassette and four transcription factors for induced pluripotent stem cells (iPSC) was constructed and transduced into ZF4 cells, and these exogenous genes were simultaneously delivered and transcribed efficiently in drug-selected ZF4 cells, proving the practicability of this modified recombinant baculovirus system. We also proved that the WSSV ie1 promoter had robust activity in fish cells in vitro and in vivo. Taken together, this modified recombinant baculovirus can be a favorable transgenic tool to obtain transient or stable transgenic fish cells.

## 1. Introduction

Originally, the baculovirus expression system, based on the *Autographa californica* multiple nucleopolyhedrovirus (AcMNPV), was mainly utilized for eukaryotic protein expression [1] or viral antigen production in host insect cells [2]. Later on, the recombinant baculovirus was used to deliver exogenous reporter genes into mammalian hepatocytes, which expanded its application in a large number of animal cells [3,4]. Today, baculovirus is widely used as a gene delivery vector for multifarious purposes due to its biological safety, nonreplication nature, low cytotoxicity, large capacity for cloning, and simplicity of operation [4]. However, the application of baculovirus as a shuttle vector or gene delivery vector has mainly focused on mammalian and avian cells. In fish cells, there have been few studies concerning baculovirus-mediated transient single-gene expression. For instance, baculovirus efficiently mediates gene delivery into medaka (*Oryzias latipes*) embryonic stem cells [5]. It also delivers the gene of interest to a particular location by injecting viruses into specific tissues directly in zebrafish (*Danio rerio*) [6]. Moreover, baculovirus can transduce differentiated cells such as *Cyprinus carpio* Epithelioma papulosum cyprini (EPC) cells [7,8]. Nevertheless, the delivery of large DNA fragments to fish cells and the establishment of stably integrated cell lines via baculovirus have not yet been reported.

It is desirable for an ideal transgenic system such as baculovirus containing robust shuttle promoter for multiple gene expression. Current baculovirus systems, such as pFastBac-Dual, contain two promoters: the polyhedrin (PH) promoter and the late 10-kDa fibrous polypeptide (P10) promoter. However, the PH promoter of baculovirus is inactive in mammalian cells [3,9], and the P10 promoter requires other AcMNPV gene products for activity [10]. Thus, the PH and P10 promoters are not suitable as shuttle promoters for recombinant baculovirus when transducing into other cells. Based on the literature review, the cytomegalovirus (CMV) promoter is most widely used as a shuttle promoter of recombinant baculovirus [3,5,11], whereas the white spot syndrome virus (WSSV) immediate-early gene 1 (ie1) (WSSV ie1) promoter can serve as a baculovirus-independent shuttle promoter between insect and mammalian cells [9]. A previous study also mentioned that the WSSV ie1 promoter is active in three fish cell lines, including 24-h postfertilization zebrafish embryo (PAC2), Chinook salmon (*Oncorhynchus tshawytscha*) embryo (CHSE-214), and EPC cells [12]. The activity of the WSSV ie1 promoter in fish cells delivered by baculovirus transduction has not been elaborated.

On account of its large DNA fragment cloning capacity, recombinant baculovirus has been widely used to transduce mammalian cells for the codelivery of multiple genes, including induced pluripotent stem cell (iPSC) factors [13], which has not been examined in fish cells. In this study, we modified a recombinant baculovirus system, which simultaneously allowed for rapid reporter assay, drug selection, and exogenous gene expression by dual-shuttle promoters, and applied it to five fish cell lines. Moreover, we tested the stable delivery capacity of large DNA fragments containing four iPSC factors to *Danio rerio* embryonic fibroblast (ZF4) cells.

## 2. Results

### 2.1. Construction of Recombinant Baculovirus Containing Dual-Shuttle Promoters Following a pr/pf Cassette

**Figure 1:**
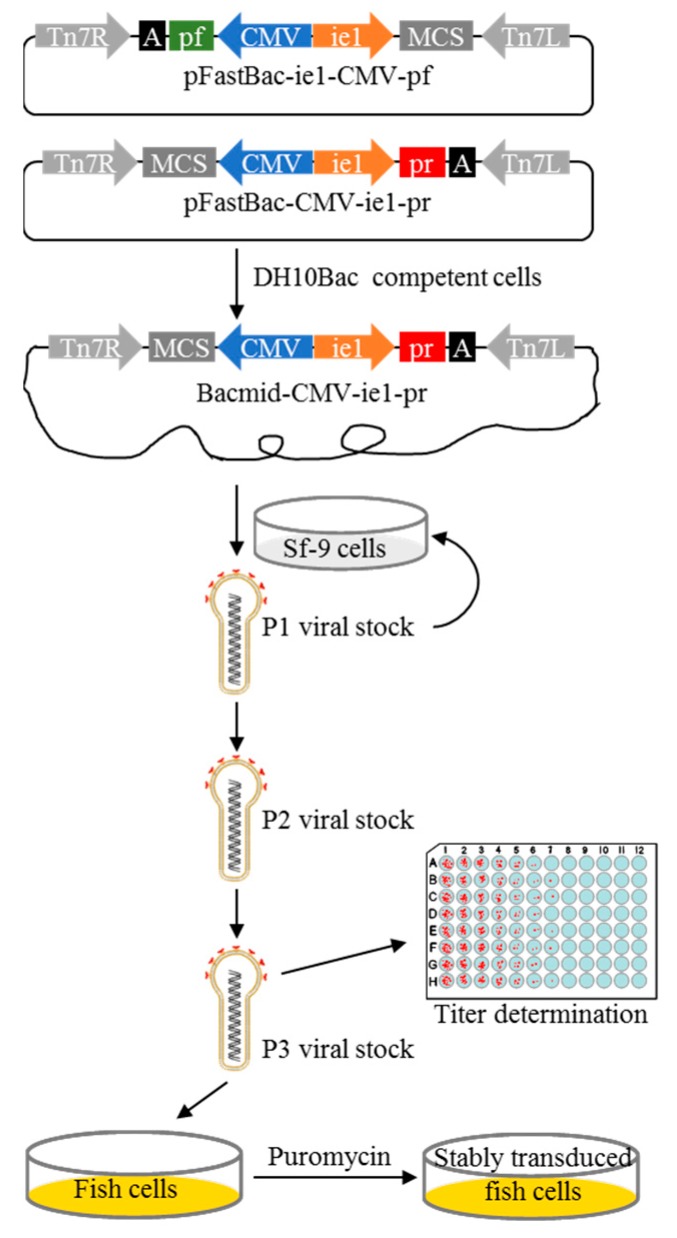
Construction of shuttle plasmid and illustration of recombinant baculovirus production and transduction of fish cells. In plasmid pFastBac-ie1-CMV-pf/pFastBac-CMV-ie1-pr, a puromycin–red fluorescent protein (Puro-RFP, *pr*) or puromycin–green fluorescent protein (Puro-GFP, *pf*) reporter cassette was driven by a cytomegalovirus (CMV) or white spot syndrome virus (WSSV) immediate-early gene 1 (ie1) (WSSV ie1) promoter, and an adjacent WSSV ie1 or CMV promoter was used to express the gene of interest in the reverse direction. The donor plasmid was transformed to DH10Bac competent cells to generate Bacmid, which was then transfected to Sf-9 cells to obtain the baculovirus. The baculoviral stock was amplified by infecting Sf-9 cells and titrated and transduced into fish cells. MCS, multiple cloning sites. A, poly(A). P1 (P2, P3) viral stock, Passage-1 (-2, -3) viral stock.

In the shuttle vectors pFastBac-CMV-ie1-pr and pFastBac-ie1-CMV-pf, the dual promoters P10 and PH of donor plasmid pFastBac-Dual were replaced by CMV and WSSV ie1 promoters to drive a gene of interest, and a puromycin–red fluorescent protein (Puro-RFP, *pr*) or puromycin–green fluorescent protein (Puro-GFP, *pf*) cassette was inserted after the WSSV ie1 or CMV promoter to act as a fluorescence reporter and drug resistance gene. Multiple cloning sites behind another promoter were used for further cloning. The recombinant baculoviruses BV-CMV-ie1-pr and BV-ie1-CMV-pf were generated subsequently and transduced to various fish cell lines (Figure 1). The detailed maps and sequences of pFastBac-CMV-ie1-pr (GenBank Accession Number: MK161518) and pFastBac-ie1-CMV-pf (GenBank Accession Number: MK161519) are shown in the Supplementary Figures S1 and S2.

### 2.2. Fast and Precise Titer Determination Based on Fluorescence Assay

**Figure 2:**
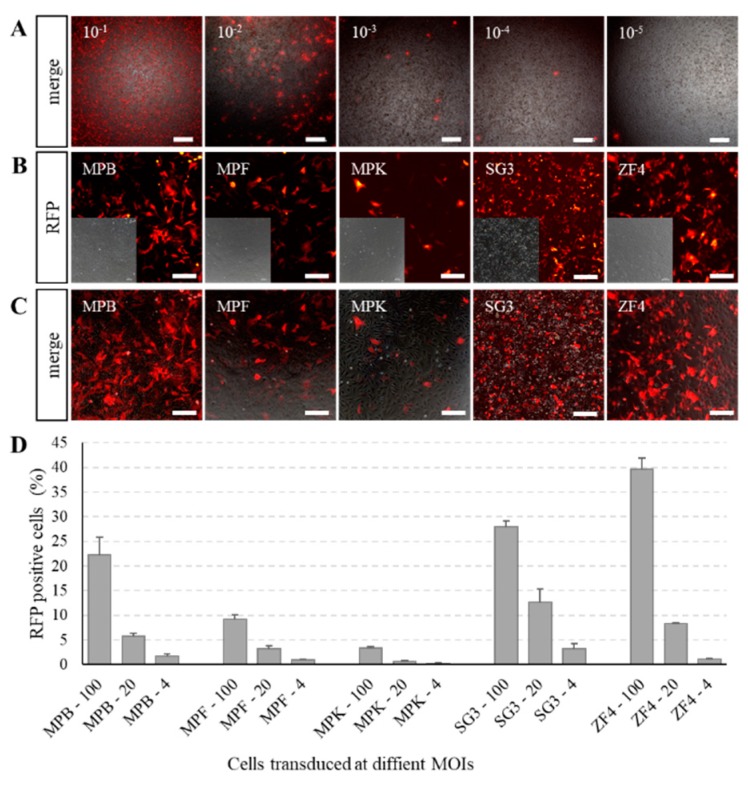
Cells transduced with recombinant baculovirus. (**A**) Titer determination by end-point dilution method based on RFP fluorescence. Baculoviral stocks diluted from 10^−2^ to 10^−8^ times with 10 μL were added to a 96-well plate. The number on the upper left corner of each image is the volume (μL) of original baculoviral stock added to each well. Scale bars, 400 μm. (**B**,**C**) Expression of RFP in *Mylopharyngodon piceus* bladder (MPB), fin (MPF), and kidney (MPK); *Oryzias latipes* spermatogonia (SG3); and *Danio rerio* embryonic fibroblast (ZF4) cells transduced with BV-CMV-ie1-pr at a multiplicity of infection (MOI) of 20. The expression of RFP was observed under an inverted fluorescence microscope after 3 days of transduction. Scale bars, 200 μm. (**D**) Transduction efficiencies of BV-CMV-ie1-pr in MPB, MPF, MPK, SG3, and ZF4 cells at different MOIs. The transduced efficiencies were determined by counting RFP-positive cells under an inverted fluorescence microscope at 3 days post-transduction in triplicate. The number after the cell name is the corresponding incubation dose in MOI. Values are indicated as mean ± SD.

The titers of modified baculovirus were quantitated accordingly. After incubation of the virus into cells for 3 days, a large number of red fluorescent plaques appeared in wells which contained the maximum dose of baculoviral stock and decreased gradually with the reduction of baculoviral stock. The red fluorescent plaques were easy to observe and distinguish (Figure 2A). After 4–7 days of incubation, there was no increase in wells which contained red fluorescence; therefore, the data of 3 days post-infection were regarded as the final results. The titer of the baculoviral stock was subsequently calculated according to the 50% tissue culture infectious dose method (TCID_50_) method. Therefore, the titer of modified baculovirus could be quantitated fast and precisely.

### 2.3. Efficiently Stable Gene Delivery into Fish Cells by Recombinant Baculovirus

To evaluate the transient transduction efficiency, five fish cell lines were tested. Red fluorescence was exhibited clearly in *Mylopharyngodon piceus* bladder (MPB), fin (MPF), and kidney (MPK); *Oryzias latipes* spermatogonia (SG3); and ZF4 cells after 3 days transduction with BV-CMV-ie1-pr (multiplicity of infection (MOI) = 20) (Figure 2B,C). Moreover, MPB, SG3, and ZF4 cells showed high densities of red fluorescence, which implied some desirable transfection efficiencies. We further measured the transduction efficiencies of recombinant baculovirus BV-CMV-ie1-pr in MPB, MPF, MPK, SG3, and ZF4 cells at different MOIs (Figure 2D). Moreover, another modified baculovirus, BV-ie1-CMV-pf, was transduced into ZF4 cells and selected by puromycin for several days (Figure 1 and Supplementary Figure S3).

**Figure 3:**
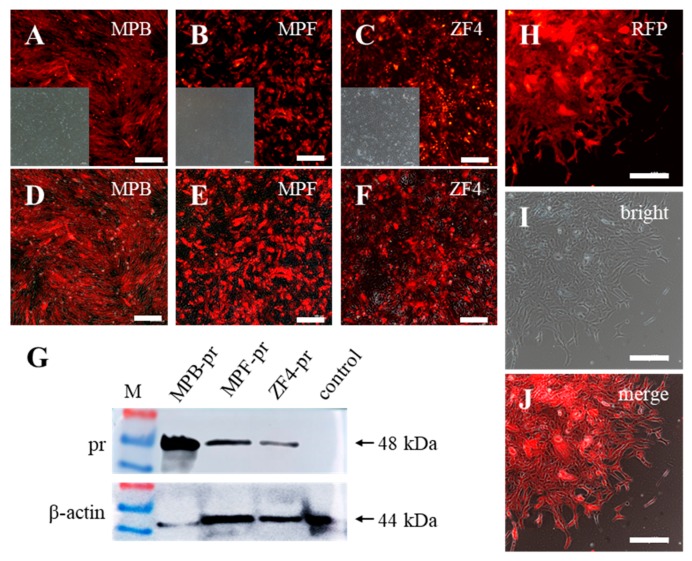
Expressions of RFP in stably integrated fish cell lines. (**A**–**F**) Expression of RFP in stable MPB, MPF, and ZF4 cells after baculovirus transduction and drug selection. (**A**–**C**) are images caught at red fluorescence and bright. (**D**–**F**) are merged images. (**G**) Western blot results of Puromycin-RFP (Pr) protein (48 kDa) in stably transduced MPB, MPF, and ZF4 and un-transduced control cell lines. M represents protein markers. (**H**–**J**) Monoclonal cell growth assay of stably transduced MPF cells. The expression of RFP was observed under an inverted fluorescence microscope. Scale bars, 200 μm.

To examine whether an exogenous gene could be stably delivered into the fish cells via our recombinant baculovirus system, MPB, MPF, and ZF4 cells were transduced with BV-CMV-ie1-pr at a MOI of 20, and then cells were cultured under a selection pressure (1 μg/mL puromycin) for about 10 passages. During the puromycin selection, cells were subcultured at a ratio of 1:3. Finally, the small portion and weak red fluorescence became strong and uniform after drug selection (Figure 3A–F). The Western blot results showed that a distinct band of approximately 48 kDa Pr protein was detected in those cells, in which the MPB cell line had maximum Pr protein expression (Figure 3G). MPF cells had a capacity of forming cell monoclones, so we further performed a monoclonal growth assay to detect the hereditary stability of the delivered exogenous gene. Before being reseeded to form the monoclones, MPF cells were transduced with BV-CMV-ie1-pr and selected with puromycin until they maintained a uniform and stable state, as described above. Cells were cultured without puromycin during the monoclonal formation assay. After 15 days of culture, all of the monoclones in the well were RFP positive, and all of the cells in each monoclone were RFP positive, too (Figure 3H–J).

### 2.4. The Capacity of Stable Delivery of Multiple Genes into Fish Cells by Recombinant Baculovirus

**Figure 4:**
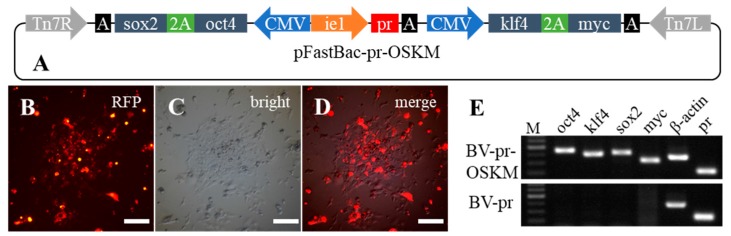
Expressions of exogenous *oct4, sox2, klf4*, and *myc* in BV-pr-OSKM-transduced ZF4 cells. (**A**) Structure of pFastBac-pr-OSKM. Driven by a CMV promoter, *oct4* and *sox2* were fused by a 2A peptide, which was similar in *klf4* and *myc*. (**B**–**D**) ZF4 cells transduced with BV-pr-OSKM. Scale bars, 200 μm. (**E**) cDNA fragments of *oct4* (400 bp), *klf4* (399 bp), *sox2* (385 bp), *myc* (312 bp), *β*-*actin* (382 bp), and *pr* (221 bp) were detected by semiquantitative RT-PCR in ZF4 cells transduced with BV-pr-OSKM or control BV-CMV-ie1-pr (BV-pr). M, DNA marker; oct4, octamer-binding transcription factor 4; sox2, sex determining region Y-box 2; klf4, Kruppel-like factor 4; myc, homology with the Avian viral gene v-myc.

To evaluate the capacity of recombinant baculovirus for DNA delivery to fish cells, we constructed four fish iPSC factors in a single plasmid. The coding sequences of four iPSC factors (*oct4* (octamer-binding transcription factor 4), *sox2* (sex determining region Y-box 2), *klf4* (Kruppel-like factor 4), and *myc* (homology with the Avian viral gene v-myc)) from *Megalobrama amblycephala* were cloned and constructed into a Bacmid frame accordingly, and the recombinant baculovirus BV-pr-OSKM contained approximate 8 kb exogenous DNA fragments (Figure 4A). Since the genome database is available in zebrafish and the pluripotent associated genes, such as *nanog*, *oct4*, and *sox2*, have been identified [14], the ZF4 cells were transduced with BV-pr-OSKM and selected by puromycin to obtain the stable transgenic cells (Figure 4B–D). After drug selection for one month and approximately 10 subcultures at a ratio of 1:3, the transcripts of four exogenous factors from transgenic cells were detected by semiquantitative RT-PCR. Though the PCR amplification was for 28 cycles, those genes could be strikingly and simultaneously detected (Figure 4E), indicating that the four exogenous genes were delivered into ZF4 cells and RNAs were also efficiently transcribed.

### 2.5. The Robust Promoter Activity of WSSV ie1 in Fish Cells

**Figure 5:**
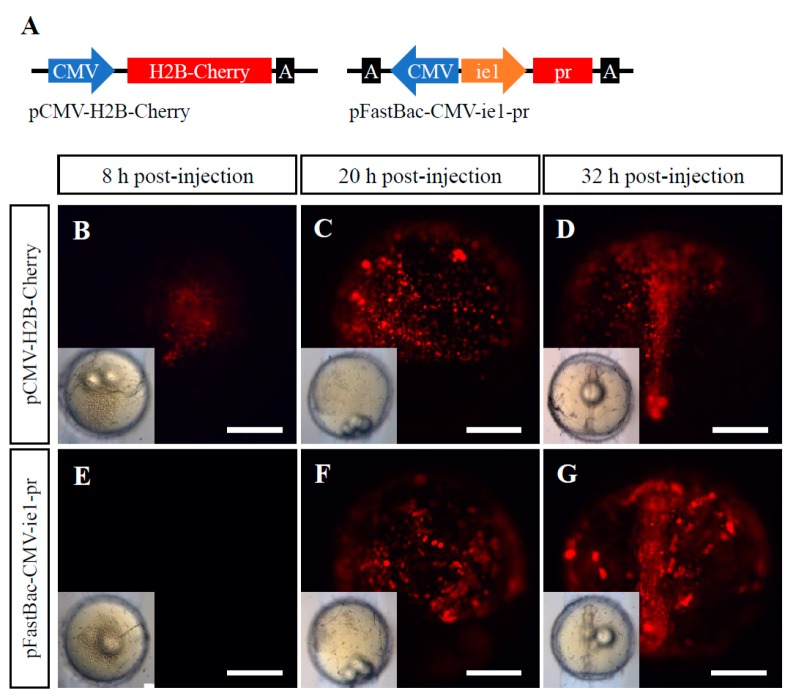
The activity of the WSSV ie1 promoter in medaka embryos. (**A**) Rough sketch of pCMV-H2B-Cherry and pFastBac-CMV-ie1-pr that were used for microinjection into fish embryos. In pCMV-H2B-Cherry, a *H2B-cherry* cassette was driven by a CMV promoter, and the H2B polypeptide was a nuclear localization signal. In pFastBac-CMV-ie1-pr, a *pr* cassette was driven by a WSSV ie1 promoter. A, poly(A). (**B**–**G**) The RFP expressions in medaka embryos where the plasmids pCMV-H2B-Cherry or pFastBac-CMV-ie1-pr were injected. Scale bar, 500 μm.

In the baculovirus BV-CMV-ie1-pr, a WSSV ie1 promoter was used to drive the *pr* cassette. Both the intense red fluorescence signals and Western blot signals of Pr protein in the above five fish cell lines implied the robust activity of the WSSV ie1 promoter in vitro in fish cells (Figure 1, Figure 2 and Figure 3). The plasmid pFastBac-CMV-ie1-pr was also injected into medaka embryos to test the activity of the WSSV ie1 promoter in fish cells in vivo, and the plasmid pCMV-H2B-Cherry containing a CMV promoter and a *H2B-Cherry* cassette was a control (Figure 5A). The H2B polypeptide is a nuclear localization signal [15], so RFP signals were concentrated in the nuclei of cells (Figure 5B–D). It was demonstrated that the WSSV ie1 promoter had a strong and universal activity in pFastBac-CMV-ie1-pr-injected medaka embryos (Figure 5F,G), which had equivalent activity to the CMV promoter in pCMV-H2B-Chery-injected embryos. In addition, we found that the WSSV ie1 promoter initiated robust activity at 18–24 h post-injection in fish cells (Figure 5F), which was slightly later compared with CMV promoter at 6–8 h (Figure 5B).

## 3. Discussion

A rapid way to generate stably integrated fish cell lines with large DNA capacity is required for fundamental research. It is generally considered that delivery of DNA into fish cells is relatively difficult when using traditional transfection reagents, and some of them are toxic to fish cells [16,17], or transfection efficiencies can vary largely among different labs. Alternative methods such as electroporation can deliver DNA into fish cells, but it is costly and requires plenty of plasmid and cell materials, so the application is still limited. Previously, there were a few attempts to transduce fish cells/origins via recombinant baculovirus [5,6,7,8], and most of them were used for transient gene delivery but were not extended for stable integration. Huang et al. [8] reported that the percentage of transduced enhanced green fluorescent protein -positive fish cells decreases dramatically during the first 15 days. In order to generate multiple-gene and stably integrated fish cell lines, we established a modified recombinant baculovirus system: driven by CMV or WSSV ie1 promoters, a *pf*/*pr* cassette was applied as a fluorescence reporter as well as the puromycin-resistant gene, and the multiple cloning sites behind another promoter were used for further cloning. The transduction efficiencies of baculovirus in different fish cell lines were rather different, as a previous study demonstrated [8]. The transduction efficiency of baculovirus in one cell line at a certain MOI is likely to be limited by the affinity between the baculovirus envelope protein and the cytomembrane of the recipient. The titer of baculoviral stock is an essential parameter during transduction because the transduction dosage in MOI is obtained from the titer, and therefore it is necessary to measure the titer of any batch of viral stock. In previous studies, various methods have been applied to quantitate recombinant baculoviruses, such as the traditional plaque assay [18], flow cytometry assay [19], immunological assay [20], MTT assay [21], alamar blue assay [21], quantitative real-time PCR [21,22], methods based on visible cell sizes [21,23], viable cell side scatter [24], etc., most of which take up to several days, require expensive detection kits, or entail multiple laborious operation steps. In this study, the titer of baculoviral stock was quantitated based on an end-point dilution method with fluorescence, which is sensitive and rapid because of the robust promotor and luminous fluorescent signals, economical due to the simple reagent, and convenient to operate, as previous studies have demonstrated [21,25,26]. Except for the MOI, there are some other factors that may influence the transduction efficiency or the determination of transduction efficiency, such as incubation time, the incubation surrounding solutions, observation time post-transduction [8], the cellular state, the activity of the promoter, and so on. The transduction efficiencies in one cell line at a certain MOI are likely to be rather different in different situations or among different labs; for example, the transduction efficiencies in SG3 cells were rather different between this study and a previous study [5].

Here, we performed a cell monoclonal formation assay to test the ability of recombinant baculovirus to stably deliver DNA into fish cells after drug selection. During the monoclonal assay, selected cells were grown under a natural condition without puromycin. All of the monoclones were RFP positive, and cells in each monoclone were RFP positive. Since baculovirus genomic DNA cannot replicate in the transduced cells [27], it was attractive to speculate that the exogenous gene was stably integrated into the chromosome of the fish cells, while Genome Walking or some other sequencing strategy might be needed to confirm the accurate genomic position where the exogenous gene was inserted.

The cloning capacity of baculovirus is as large as 38 kb [28], which is much longer than that of the retrovirus *Tol2* transposon or the *Sleeping Beauty* transposon [29,30,31]. In this study, approximately 8-kb exogenous DNA fragments was efficiently delivered to fish cells, indicating that baculovirus can serve as a powerful gene-transfer tool for multiple genes into fish cells synchronously. By using BV-pr-OSKM baculovirus, four iPSC factors (*oct4*, *sox2*, *klf4*, and *myc*) were delivered in fish cells and were efficiently transcribed. Thus, this transgenic tool is favorable for fish iPSCs research in the future.

In the present study, we took advantage of the WSSV ie1 promoter, which has not been commonly used in fish cells before. Our results demonstrated that the WSSV ie1 promoter has robust activity in different kinds of fish cells and medaka embryos. A previous study proved that the WSSV ie1 promoter is more efficient than the CMV promoter in PAC2 cells [12], has an intense activity in avian [32] and mammalian cells [9], and shows stronger activity than the CMV promoter in Sf-9 cells or the baculovirus early-to-late (ETL) promoter in HeLa, BHK21, and Vero cells, respectively [9,32]. Therefore, the WSSV ie1 promoter can act as a powerful shuttle promoter between insect cells and other eukaryotic cells.

In conclusion, our modified recombinant baculovirus system allows for easy observation, fast titer determination, rapid drug selection, and transient or stable multiple-gene delivery, which is likely to be appropriate in any kind of eukaryotic cell for gene delivery manipulation.

## 4. Materials and Methods

### 4.1. Cell Culture

Insect fall armyworm (*Spodoptera frugiperda*) (Sf-9) cells, kindly provided by Prof ShengBo Cao, HZAU, were cultured in Grace’s Insect Cell Culture Medium (Gibco, Carlsbad, CA, USA) supplemented with 10% fetal bovine serum (FBS) (Gibco, Carlsbad, CA, USA). Black carp *(Mylopharyngodon piceus)* kidney (MPK) cells [33], bladder (MPB) cells, and fin (MPF) cells were derived from our lab and cultured in ESM4 medium supplemented with 10% FBS (GEMINI, West Sacramento, CA, USA) [34]; medaka spermatogonial (SG3) cells [35], provided by Prof. Yunhan Hong, National University of Singapore, were cultured in ESM4 medium supplemented with 15% FBS (Gibco, Carlsbad, CA, USA); and zebrafish embryonic fibroblast (ZF4) cells [36], maintained in our lab, were cultured in DMEM/F12 medium containing 10% FBS with 3.5% CO_2_. All cells were cultured at 28 °C.

### 4.2. Plasmid and Recombinant Baculoviral Vectors

Reporter plasmids pCVpr and pCVpf were kindly gifted by Prof. Yunhan Hong [37]. The plasmid pFastBac-Dual (Invitrogen, Carlsbad, CA, USA) was used to generate recombinant baculoviral vectors. A DNA fragment of 501-bp containing WSSV ie1 promoter [38,39] was PCR amplified from WSSV genomic DNA (GenBank Accession Number: AF369029.2) by using primers PIE1F/ PIE1R and cloned into pFastBac-Dual with *Xho* I/*Eco*R I to generate pFastBac-ie1. To construct pFastBac-CMV-ie1-pr, a *pr* cassette from pCVpr was cloned into the *Eco*R I/*Not* I sites of pFastBac-ie1, and a CMV promoter was PCR amplified from pCVpf by using primers PMF/PMR and cloned into the *Xho* I site. To construct pFastBac-ie1-CMV-pf, the CMV promoter together with a *pf* cassette was PCR amplified from pCVpf by using primers PPF/PPR and cloned into the *Kpn* I/*Xho* I of pFastBac-ie1 (Figure 1).

To construct the iPSC factor vector, the open reading frame of four fish iPSC factors (*oct4*, *sox2*, *klf4*, and *myc*—GenBank Accession Number: KY994571, KY994572, KY994573, and KY994574, respectively) were cloned from cDNA libraries of blunt snout bream *(Megalobrama amblycephala)* and then inserted into pFastBac-CMV-ie1-pr to form pFastBac-pr-OSKM, in which *oct4* and *sox2* were fused by a 2A peptide [40] and driven by a CMV promoter as well as *klf4* and *myc* fusion cassette (Figure 4A). Briefly, *oct4* and *sox2* were PCR amplified using two specific primer pairs (Oct4F/Oct4R, Sox2F/Sox2R) and inserted into the *Xho* I/*Kpn* I of pFastBac-CMV-Ie1-pr by In-Fusion Cloning (Clontech, Mountain View, CA, USA) to construct vector pFastBac-CMV-ie1-pr-oct4-sox2. The CMV promoter fragment and *klf4* were amplified by using two specific primer pairs (CMVF/CMVR, Klf4F/Klf4R) and inserted into the *Nde* I/*Eco*R I of pIRES2-myc by In-Fusion Cloning to generate the vector containing a CMV-klf4-myc fragment. Then, the CMV-klf4-myc fragment was embedded in the *Not* I/*Hin*d III sites of pFastBac-CMV-ie1-pr-oct4-sox2 to construct iPSC vector pFastBac-pr-OSKM (GenBank Accession Number: MK161520). Details of primers and PCR conditions are indicated in the Supplementary Table.

The recombinant baculoviruses BV-CMV-ie1-pr, BV-ie1-CMV-pf, and BV-pr-OSKM were generated and amplified using the Bac-to-Bac system (Invitrogen, Carlsbad, CA, USA). The donor plasmids pFastBac-CMV-ie1-pr, pFastBac-ie1-CMV-pf, and pFastBac-pr-OSKM were isolated and transformed to DH10Bac competent cells. Positive transformants were detected by blue-white selection and bacterial PCR. Bacmids were isolated using a BAC/PAC Isolation Kit (OMEGA Bio-tek, Norcross, GA, USA) and transfected to Sf-9 cells, and the Passage-1 viral stocks were generated at about 4 days post-transfection. The Passage-2 baculoviruses were propagated by infecting Sf-9 cells at a MOI of 0.1 until the Passage-3 viral stocks were generated (Figure 1). Viruses were harvested by centrifugation at 1000× *g* for 5 min and then filtrated through a 0.22-μm syringe filter.

### 4.3. Titer Determination

Titers of recombinant baculoviral stocks were evaluated by an end-point dilution method based on RFP or GFP fluorescence. Briefly, 10 μL of baculoviral stocks diluted from 10^−2^ to 10^−8^ were added to 100 μL of 2 × 10^5^ cell/mL Sf-9 cells, which were seeded previously into a 96-well plate. Plates were incubated at 28 °C for 3 days, and the RFP or GFP fluorescence of each well was monitored by using a Nikon ECLIPSE Ti inverted fluorescence microscope (Nikon, Tokyo, Japan). The titer of recombinant baculovirus was calculated according to the TCID_50_ [41]. The TCID_50_ was converted to plaque forming units/μL (pfu/μL) for the convenience of calculating the MOI values during transduction.

### 4.4. Transduction of Fish Cells

Fish cells were seeded at 1–3 × 10^5^ cells/mL into 12-well plates (70%–80% confluence). Culture medium was removed and replaced with baculoviral stock complementing with basal cell culture medium or Opti-MEM. Cells were incubated overnight at 28 °C, and then virus inoculum was replaced with fresh medium. MOIs varied with the volume of baculoviral stock and fish cell numbers. For example, the TCID_50_ of one stock of baculovirus BV-CMV-ie1-pr P3 was 10^5.75^/μL (5.6 × 10^5^/μL), thus the pfu/μL value of this baculovirus stock was 4 × 10^5^/μL. Accordingly, 10 μL of baculoviral stock was added into 2 × 10^5^ fish cells at a MOI of 20. After 2–3 days of incubation, fish cells were observed for RFP or GFP expression using an inverted fluorescence microscopy.

### 4.5. Fluorescence-Positive Cell Counting

At 3 days post-transduction, cells were trypsinized and suspended in PBS. The positive red/green fluorescence cells and total cells in three different visual fields were counted with a hematocytometer under an inverted fluorescence microscope, and the percentage of positive red/green fluorescence cells was calculated. Each treatment had three repetitions, and the data were represented as mean ± SD.

### 4.6. Western Blot

Cells were trypsinized and washed twice with PBS, and cellular protein was extracted using a Mammalian Protein Extraction Kit (CWBIO, Beijing, China). The concentration of cellular protein was measured by Quick Start Bradford protein assay (Bio-Rad, Hercules, CA, USA). Cellular proteins were electrophoresed and transferred onto a Hybond-PVDF membrane (GE, Boston, MA, USA). The membrane was firstly incubated for 1 h at room temperature in blocking buffer (TBST containing 5% skim milk) and then incubated overnight at 4 °C with a mouse monoclonal primary antibody against RFP (Abbkine, Wuhan, China; 1:1000 dilutions). The membrane was washed with TBST and incubated with a horseradish peroxidase-conjugated secondary antibody (Vazyme, Nanjing, China; 1:10000 dilutions) for 1 h at room temperature. After washing, the membrane was stained using the Western ECL Substrate (Bio-Rad, Hercules, CA, USA). The protein bands were visualized by a Biomolecular Imager (GE, Boston, MA, USA). Then, the same Hybond-PVDF membrane was washed in stripping buffer (CWBIO, Beijing, China) for 30 min, reprobed by an antibody against β-actin (Vazyme, Nanjing, China; 1:1000 dilutions), and then processed as mentioned above.

### 4.7. Colony Formation Assay

The transduced cells were selected with 1 μg/mL puromycin for about 10 passages, and cells were usually subcultured into a new well or flask at a ratio of 1:3 within 3 days. Stably transduced fish cells were diluted to 1 × 10^2^ cells/mL with culture medium without puromycin and seeded into a 12-well plate. Cells were observed for RFP expression using an inverted fluorescence microscopy after 15 days of culture.

### 4.8. PCR Assay

Cellular RNA was extracted by using the RNAiso Plus reagent (Takara, Shiga, Japan). First-strand cDNA was synthesized by using the PrimeScript™ RT reagent Kit with gDNA Eraser (Takara, Shiga, Japan). PCR was performed using four specific primer pairs (Oct4-F/Oct4-R, Sox2-F/Sox2-R, Klf4-F/Klf4-R, and Myc-F/Myc-R). As a positive control, *β-actin* (GenBank Accession Number: NM_001104808) and *pr* were amplified from the same set of cDNA samples using gene-specific primers (Actin-F/Actin-R, Pr-F/Pr-R). All the PCR amplifications were run in a 20-μL volume containing 10 ng of cDNA reaction for 28 cycles (25 s at 94 °C, 25 s at 60 °C, and 20 s at 72 °C) for all genes. Details of primers and PCR conditions are indicated in the Supplementary Table.

### 4.9. Microinjection of Medaka Embryos

Microinjection and embryo culture were carried out as previously described [42]. Briefly, 2–4 nL of 30 ng/μL plasmid and 0.2% phenol red were injected into the cytoplasm of fertilized medaka embryos at one-cell stage. Plasmids used for microinjection are indicated in Figure 5A. The expression of red fluorescence protein in embryos was observed under a fluorescence stereo microscope (Leica, Wetzlar, German).

## Supplementary Materials

Supplementary materials can be found at http://www.mdpi.com/1422-0067/19/12/3767/s1.
