# Supplementary material for: Efficient and Stable Delivery of Multiple Genes to Fish Cells by a Modified Recombinant Baculovirus System
Source: Int J Mol Sci. 2018 Nov 27;19(12):3767. doi: 10.3390/ijms19123767 (PMC6321422; doi:10.3390/ijms19123767)
Supplement: Supplementary file 1 [file ijms-19-03767-s001.pdf]

Supplementary Figure 1

Detailed map and sequence of modified plasmid pFastBac-CMV-ie1-pr.

multiple cloning site

CMV enhancer + CMV promoter

WSSV ie1 promoter

PuroR (puromycin N-acetyltransferase)

DsRed

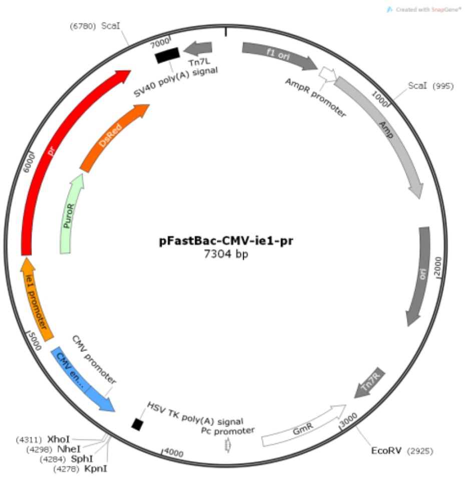

ttctctgtcacagaatgaaaatttttctgtcatctcttcgttattaatgtttgtaattgactgaatatcaacgcttatttgcagcctgaatggcgaatgggacgcgccctgtagcggcgcattaagcgc

ggcgggtgtggtggttacgcgcagcgtgaccgctacacttgccagcgccctagcgcccgctcctttcgctttcttcccttcctttctcgccacgttcgccggctttccccgtcaagctctaaatc

gggggctccctttagggttccgatttagtgctttacggcacctcgaccccaaaaaacttgattagggtgatggttcacgtagtgggccatcgccctgatagacggtttttcgccctttgacgttgg

agtccacgttctttaatagtggactcttgttccaaactggaacaacactcaaccctatctcggtctattcttttgatttataagggattttgccgatttcggcctattggttaaaaaatgagctgatttaa

caaaaatttaacgcgaattttaacaaaatattaacgtttacaatttcaggtggcacttttcggggaaatgtgcgcggaacccctatttgtttatttttctaaatacattcaaatatgtatccgctcatgag

acaataaccctgataaatgcttcaataatattgaaaaaggaagagtatgagtattcaacatttccgtgtcgcccttattcccttttttgcggcattttgccttcctgtttttgctcacccagaaacgctg

gtgaaagtaaaagatgctgaagatcagttgggtgcacgagtgggttacatcgaactggatctcaacagcggtaagatccttgagagttttcgccccgaagaacgttttccaatgatgagcactt

ttaaagttctgctatgtggcgcggtattatcccgtattgacgccgggcaagagcaactcggtcgccgcatacactattctcagaatgacttggttgagtactcaccagtcacagaaaagcatctt

acggatggcatgacagtaagagaattatgcagtgctgccataaccatgagtgataacactgcggccaacttacttctgacaacgatcggaggaccgaaggagctaaccgcttttttgcacaa

catgggggatcatgtaactcgccttgatcgttgggaaccggagctgaatgaagccataccaaacgacgagcgtgacaccacgatgcctgtagcaatggcaacaacgttgcgcaaactatta

actggcgaactacttactctagcttcccggcaacaattaatagactggatggaggcggataaagttgcaggaccacttctgcgctcggcccttccggctggctggtttattgctgataaatctgg

agccggtgagcgtgggtctcgcggtatcattgcagcactggggccagatggtaagccctcccgtatcgtagttatctacacgacggggagtcaggcaactatggatgaacgaaatagacag

atcgctgagataggtgcctcactgattaagcattggtaactgtcagaccaagtttactcatatatactttagattgatttaaaacttcatttttaatttaaaaggatctaggtgaagatcctttttgataat

ctcatgaccaaaatcccttaacgtgagttttcgttccactgagcgtcagaccccgtagaaaagatcaaaggatcttcttgagatcctttttttctgcgcgtaatctgctgcttgcaaacaaaaaaac

caccgctaccagcggtggtttgtttgccggatcaagagctaccaactctttttccgaaggtaactggcttcagcagagcgcagataccaaatactgtccttctagtgtagccgtagttaggccac

cacttcaagaactctgtagcaccgcctacatacctcgctctgctaatcctgttaccagtggctgctgccagtggcgataagtcgtgtcttaccgggttggactcaagacgatagttaccggataa

ggcgcagcggtcgggctgaacggggggttcgtgcacacagcccagcttggagcgaacgacctacaccgaactgagatacctacagcgtgagcattgagaaagcgccacgcttcccgaa

gggagaaaggcggacaggtatccggtaagcggcagggtcggaacaggagagcgcacgagggagcttccagggggaaacgcctggtatctttatagtcctgtcgggtttcgccacctctg

acttgagcgtcgatttttgtgatgctcgtcaggggggcggagcctatggaaaaacgccagcaacgcggcctttttacggttcctggccttttgctggccttttgctcacatgttctttcctgcgttat

cccctgattctgtggataaccgtattaccgcctttgagtgagctgataccgctcgccgcagccgaacgaccgagcgcagcgagtcagtgagcgaggaagcggaagagcgcctgatgcgg

tattttctccttacgcatctgtgcggtatttcacaccgcagaccagccgcgtaacctggcaaaatcggttacggttgagtaataaatggatgccctgcgtaagcgggtgtgggcggacaataaa

gtcttaaactgaacaaaatagatctaaactatgacaataaagtcttaaactagacagaatagttgtaaactgaaatcagtccagttatgctgtgaaaaagcatactggacttttgttatggctaaag

caaactcttcattttctgaagtgcaaattgcccgtcgtattaaagaggggcgtggccaagggcatggtaaagactatattcgcggcgttgtgacaatttaccgaacaactccgcggccgggaa

gccgatctcggcttgaacgaattgttaggtggcggtacttgggtcgatatcaaagtgcatcacttcttcccgtatgcccaactttgtatagagagccactgcgggatcgtcaccgtaatctgcttg

cacgtagatcacataagcaccaagcgcgttggcctcatgcttgaggagattgatgagcgcggtggcaatgccctgcctccggtgctcgccggagactgcgagatcatagatatagatctca

ctacgcggctgctcaaacctgggcagaacgtaagccgcgagagcgccaacaaccgcttcttggtcgaaggcagcaagcgcgatgaatgtcttactacggagcaagttcccgaggtaatcg

gagtccggctgatgttgggagtaggtggctacgtctccgaactcacgaccgaaaagatcaagagcagcccgcatggatttgacttggtcagggccgagcctacatgtgcgaatgatgccca

tacttgagccacctaactttgttttagggcgactgccctgctgcgtaacatcgttgctgctgcgtaacatcgttgctgctccataacatcaaacatcgacccacggcgtaacgcgcttgctgcttg

gatgcccgaggcatagactgtacaaaaaaacagtcataacaagccatgaaaaccgccactgcgccgttaccaccgctgcgttcggtcaaggttctggaccagttgcgtgagcgcatacgct

acttgcattacagtttacgaaccgaacaggcttatgtcaactgggttcgtgccttcatccgtttccacggtgtgcgtcacccggcaaccttgggcagcagcgaagtcgaggcatttctgtcctgg

ctggcgaacgagcgcaaggtttcggtctccacgcatcgtcaggcattggcggccttgctgttcttctacggcaaggtgctgtgcacggatctgccctggcttcaggagatcggtagacctcg
gccgtcgcggcgcttgccggtggtgctgaccccggatgaagtggttcgcatcctcggttttctggaaggcgagcatcgtttgttcgcccaggactctagctatagttctagtggttggcctacg
tacccgtagtggctatggcagggcttgccgccccgacgttggctgcgagccctgggccttcacccgaacttggggggttggggtggggaaaaggaagaaacgcgggcgtattggtcccaa
tggggtctcggtggggtatcgacagagtgccagccctgggaccgaaccccgcgtttatgaacaaacgacccaacacccgtgcgttttattctgtctttttattgccgtcatagcgcgggttcctt
ccggtattgtctccttccgtgtttcagttagcctcccccatctcccggtaccgcatgctatgcatcagctgctagcaccatggctcgaggatctgacggttcactaaacgagctctgcttatataga
cctcccaccgtacacgcctaccgcccatttgcgtcaacggggcggggttattacgacattttggaaagtcccgttgattttggtgccaaaacaaactcccattgacgtcaatggggtggagact
tggaaatccccgtgagtcaaaccgctatccacgcccattggtgtactgccaaaaccgcatcaccatggtaatagcgatgactaatacgtagatgtactgccaagtaggaaagtcccgtaaggt
catgtactgggcataatgccaggcgggccatttaccgtcattgacgtcaatagggggcggacttggcatatgatacacttgatgtactgccaagtgggcagtttaccgtaaatactccacccat
tgacgtcaatggaaagtccctattggcgttactatgggaacatacgtcattattgacgtcaatgggcgggggtcgttgggcggtcagccaggcgggccatttaccgtaagttatgtaacgcgg
aactccatatatgggctatgaactaatgaccccgtaattgattactattaataactagagctctgtggctaatggagaattgtcgtgccagttgtatcagcgaagcggagatgtcgggttgggcgt
gcccttcaatattgcatcatactctcttctgactcatctgatggccagtatggtgggtctaaaaccgggagagtttatcctcactcttggtgacgcacacatttataatacccacattgaggtgttaa
agaagcagttgtgccgcgtccctagaccattccctaagttgaggattttaatggctccagaaaaaattgaggactttactatcgacatgttttatcttgaggggtatcaaccacacagtggaaact
tgcagatgaaaatggctgtttgaatcatgttaaggaatttccttgttactcatttattcctagaaatggtgtaatcgctgttgtgggcggagcatatttgtgtatataagagcccgtgttagctcctcga
ttcagtcacaagagcgcacacacacgcttataactagctctctctccactcaaggaattcgccaccatgaccgagtacaagcccacggtgcgcctcgccacccgcgacgacgtccccgg
gccgtacgcaccctcgccgccgcgttcgccgactaccccgccacgcgccacaccgtcgacccggaccgccacatcgagcgggtcaccgagctgcaagaactcttcctcacgcgcgtcg
ggctcgacatcggcaaggtgtgggtcgcggacgacggcgccgcggtggcggtctggaccacgccggagagcgtcgaagcgggggcggtgttcgccgagatcggcccgcgcatggc
cgagttgagcggttcccggctggccgcgcagcaacagatggaaggcctcctggcgccgcaccggcccaaggagcccgcgtggttcctggccaccgtcggcgtctcgcccgaccacca
gggcaagggtctgggcagcgccgtcgtgctccccggagtggaggcggccgagcgcgccggggtgcccgccttcctggagacctccgcgccccgcaacctccccttctacgagcggct
cggcttcaccgtcaccgccgacgtcgaggtgcccgaaggaccgcgcacctggtgcatgacccgcaagcccggtgccactagtcgggatccaccggtcgccaccatggtgcgctcctcc
aagaacgtcatcaaggagttcatgcgcttcaaggtgcgcatggagggcaccgtgaacggccacgagttcgagatcgagggcgagggcgagggccgcccctacgagggccacaacacc
gtgaagctgaaggtgaccaagggcggccccctgcccttcgcctgggacatcctgtcccccagttccagtacggctccaaggtgtacgtgaagcaccccgccgacatccccgactacaag
aagctgtccttccccgagggcttcaagtgggagcgcgtgatgaacttcgaggacggcggcgtggtgaccgtgacccaagactcctccctgcaggacggctgcttcatctacaaggtgaagt
tcatcggcgtgaacttcccctccgacggccccgtaatgcagaagaagaccatgggctgggaggcctccaccgagcgcctgtaccccgcgacggcgtgctgaagggcgagatccacaa
ggccctgaagctgaaggacggcggccactacctggtggagttcaagtccatctacatggccaagaagcccgtgcagctgcccggctactactacgtggactccaagctggacatcacctc
ccacaacgaggactacaccatcgtggagcagtacgagcgcaccgagggccgccaccacctgttcctgtagcggccgctttcgaatctagagcctgcagtctcgacaagcttgtcgagaag
tactagaggatcataatcagccataccacatttgtagaggttttacttgctttaaaaaacctcccacacctccccctgaacctgaaacataaaatgaatgcaattgttgttgttaacttgtttattgca
gcttataatggttacaaataaagcaatagcatcacaaatttcacaaataaagcatttttttcactgcattctagttgtggtttgtccaaactcatcaatgtatcttatcatgtctggatctgatcactgctt
gagcctaggagatccgaaccagataagtgaaatctagttccaaactattttgtcatttttaattttcgtattagcttacgacgctacacccagttcccatctattttgtcactcttccctaaataatcctta
aaaactccatttccacccctcccagttcccaactattttgtccgcccacagcggggcatttttcttcctgttatgtttttaatcaaacatcctgccaactccatgtgacaaaccgtcatcttcggctact
tt

Supplementary Figure 2

Detailed map and sequence of modified plasmid pFastBac-ie1-CMV-pf.

EGFP

PuroR (puromycin N-acetyltransferase)

CMV enhancer + CMV promoter

WSSV ie1 promoter

multiple cloning site

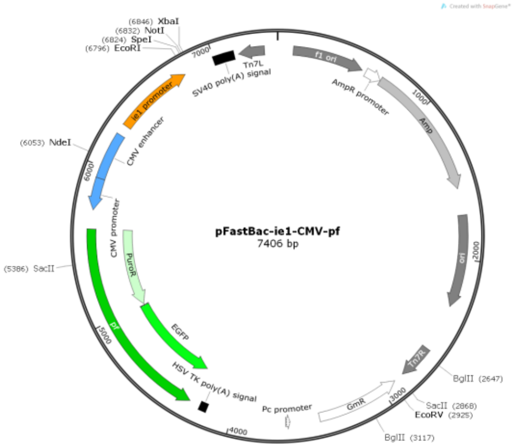

ttctctgtcacagaatgaaaatttttctgtcatctcttcgttattaatgtttgtaattgactgaatatcaacgcttatttgcagcctgaatggcgaatgggacgcgccctgtagcggcgcattaagcgc
ggcgggtgtggtggttacgcgcagcgtgaccgctacacttgccagcgccctagcgcccgctcctttcgctttcttcccttcctttctcgccacgttcgccggctttccccgtcaagctctaaatc
gggggctccctttagggttccgatttagtgctttacggcacctcgaccccaaaaaacttgattagggtgatggttcacgtagtgggccatcgccctgatagacggtttttcgccctttgacgttgg
agtccacgttctttaatagtggactcttgttccaaactggaacaacactcaaccctatctcggtctattcttttgatttataagggattttgccgatttcggcctattggttaaaaaatgagctgatttaa
caaaaatttaacgcgaattttaacaaaatattaacgtttacaatttcaggtggcacttttcggggaaatgtgcgcggaacccctatttgtttatttttctaaatacattcaaatatgtatccgctcatgag
acaataaccctgataaatgcttcaataatattgaaaaaggaagagtatgagtattcaacatttccgtgtcgcccttattcccttttttgcggcattttgccttcctgtttttgctcacccagaaacgctg
gtgaaagtaaaagatgctgaagatcagttgggtgcacgagtgggttacatcgaactggatctcaacagcggtaagatccttgagagttttcgccccgaagaacgttttccaatgatgagcactt
ttaaagttctgctatgtggcgcggtattatcccgtattgacgccgggcaagagcaactcggtcgccgcatacactattctcagaatgacttggttgagtactcaccagtcacagaaaagcatctt
acggatggcatgacagtaagagaattatgcagtgctgccataaccatgagtgataacactgcggccaacttacttctgacaacgatcggaggaccgaaggagctaaccgcttttttgcacaa
catgggggatcatgtaactcgccttgatcgttgggaaccggagctgaatgaagccataccaaacgacgagcgtgacaccacgatgcctgtagcaatggcaacaacgttgcgcaaactatta
actggcgaactacttactctagcttcccggcaacaattaatagactggatggaggcggataaagttgcaggaccacttctgcgctcggcccttccggctggctggtttattgctgataaatctgg
agccggtgagcgtgggtctcgcggtatcattgcagcactggggccagatggtaagccctcccgtatcgtagttatctacacgacggggagtcaggcaactatggatgaacgaaatagacag
atcgctgagataggtgcctcactgattaagcattggtaactgtcagaccaagtttactcatatatactttagattgatttaaaacttcatttttaatttaaaaggatctaggtgaagatcctttttgataat
ctcatgaccaaaatcccttaacgtgagttttcgttccactgagcgtcagaccccgtagaaaagatcaaaggatcttcttgagatcctttttttctgcgcgtaatctgctgcttgcaaacaaaaaaac
caccgctaccagcggtggtttgtttgccggatcaagagctaccaactctttttccgaaggtaactggcttcagcagagcgcagataccaaatactgtccttctagtgtagccgtagttaggccac
cacttcaagaactctgtagcaccgcctacatacctcgctctgctaatcctgttaccagtggctgctgccagtggcgataagtcgtgtcttaccgggttggactcaagacgatagttaccggataa
ggcgcagcggtcgggctgaacggggggttcgtgcacacagcccagcttggagcgaacgacctacaccgaactgagatacctacagcgtgagcattgagaaagcgccacgcttcccgaa
gggagaaaggcggacaggtatccggtaagcggcagggtcggaacaggagagcgcacgagggagcttccagggggaaacgcctggtatctttatagtcctgtcgggtttcgccacctctg
acttgagcgtcgatttttgtgatgctcgtcaggggggcggagcctatggaaaaacgccagcaacgcggcctttttacggttcctggccttttgctggccttttgctcacatgttctttcctgcgttat
cccctgattctgtggataaccgtattaccgcctttgagtgagctgataccgctcgccgcagccgaacgaccgagcgcagcgagtcagtgagcgaggaagcggaagagcgcctgatgcgg
tattttctccttacgcatctgtgcggtatttcacaccgcagaccagccgcgtaacctggcaaaatcggttacggttgagtaataaatggatgccctgcgtaagcgggtgtgggcggacaataaa
gtcttaaactgaacaaaatagatctaaactatgacaataaagtcttaaactagacagaatagttgtaaactgaaatcagtccagttatgctgtgaaaaagcatactggacttttgttatggctaaag
caaactcttcattttctgaagtgcaaattgcccgtcgtattaaagaggggcgtggccaagggcatggtaaagactatattcgcggcgttgtgacaatttaccgaacaactccgcggccgggaa
gccgatctcggcttgaacgaattgttaggtggcggtacttgggtcgatatcaaagtgcatcacttcttcccgtatgcccaactttgtatagagagccactgcgggatcgtcaccgtaatctgcttg
cacgtagatcacataagcaccaagcgcgttggcctcatgcttgaggagattgatgagcgcggtggcaatgccctgcctccggtgctcgccggagactgcgagatcatagatatagatctca
ctacgcggctgctcaaacctgggcagaacgtaagccgcgagagcgccaacaaccgcttcttggtcgaaggcagcaagcgcgatgaatgtcttactacggagcaagttcccgaggtaatcg
gagtccggctgatgttgggagtaggtggctacgtctccgaactcacgaccgaaaagatcaagagcagcccgcatggatttgacttggtcagggccgagcctacatgtgcgaatgatgccca
tacttgagccacctaactttgttttagggcgactgccctgctgcgtaacatcgttgctgctgcgtaacatcgttgctgctccataacatcaaacatcgacccacggcgtaacgcgcttgctgcttg
gatgcccgaggcatagactgtacaaaaaaacagtcataacaagccatgaaaaccgccactgcgccgttaccaccgctgcgttcggtcaaggttctggaccagttgcgtgagcgcatacgct
acttgcattacagtttacgaaccgaacaggcttatgtcaactgggttcgtgccttcatccgtttccacggtgtgcgtcacccggcaaccttgggcagcagcgaagtcgaggcatttctgtcctgg

ctggcgaacgagcgcaaggtttcggtctccacgcatcgtcaggcattggcggccttgctgttcttctacggcaaggtgctgtgcacggatctgccctggcttcaggagatcggtagacctcg
gccgtcgcggcgcttgccggtggtgctgaccccggatgaagtggttcgcatcctcggttttctggaaggcgagcatcgtttgttcgcccaggactctagctatagttctagtggttggcctacg
tacccgtagtggctatggcagggcttgccgccccgacgttggctgcgagccctgggccttcacccgaacttggggggttggggtggggaaaaggaagaaacgcgggcgtattggtcccaa
tggggtctcggtggggtatcgacagagtgccagccctgggaccgaaccccgcgtttatgaacaaacgacccaacacccgtgcgttttattctgtctttttattgccgtcatagcgcgggttcctt
ccggtattgtctccttccgtgtttcagttagcctcccccatctcccgctttacttgtacagctcgtccatgccgagagtgatcccggcggcggtcacgaactccagcaggaccatgtgatcgcgc
ttctcgttggggtctttgctcagggcggactgggtgctcaggtagtggttgtcgggcagcagcacggggccgtcgccgatgggggtgttctgctggtagtggtcggcgagctgcacgctgc
cgtcctcgatgttgtggcggatcttgaagttcaccttgatgccgttcttctgcttgtcggccatgatatagacgttgtggctgttgtagttgtactccagcttgtgccccaggatgttgccgtcctcct
tgaagtcgatgcccttcagctcgatgcggttcaccagggtgtcgccctcgaacttcacctcggcgcgggtcttgtagttgccgtcgtccttgaagaagatggtgcgctcctggacgtagccttc
gggcatggcggacttgaagaagtcgtgctgcttcatgtggtcggggtagcggctgaagcactgcacgccgtaggtcagggtggtcacgagggtgggccagggcacgggcagcttgccg
gtggtgcagatgaacttcagggtcagcttgccgtaggtggcatcgccctcgccctcgccggacacgctgaacttgtggccgtttacgtcgccgtccagctcgaccaggatgggcaccaccc
cggtgaacagctcctcgcccttgctcaccatgctagtggcaccgggcttgcgggtcatgcaccaggtgcgcggtccttcgggcacctcgacgtcggcggtgacggtgaagccgagccgct
cgtagaaggggaggttgcggggcgcggaggtctccaggaaggcgggcaccccggcgcgctcggccgcctccactccggggagcacgacggcgctgcccagacccttgccctggtg
gtcgggcgagacgccgacggtggccaggaaccacgcgggctccttgggccggtgcggcgccaggaggccttccatctgttgctgcgcggccagccgggaaccgctcaactcggccat
gcgcgggccgatctcggcgaacaccgcccccgcttcgacgctctccggcgtggtccagaccgccaccgcggcgccgtcgtccgcgacccacaccttgccgatgtcgagcccgacgcg
cgtgaggaagagttcttgcagctcggtgacccgctcgatgtggcggtccgggtcgacggtgtggcgcgtggcggggtagtcggcgaacgcggcggcgagggtgcgtacggcccggg
ggacgtcgtcgcgggtggcgaggcgcaccgtgggcttgtactcggtcatggtgcggccgggctgcagcttaagttcgagactgttgtgtcagaagcactgactgcgttagcaatttaactgt
gataaactaccgcaataaagcttctagtgatctgacggttcactaaacgagctctgcttatatagacctcccaccgtacacgcctaccgcccatttgcgtcaacggggcggggttattacgaca
ttttggaaagtcccgttgattttggtgccaaaacaaactcccattgacgtcaatggggtggagacttggaaatccccgtgagtcaaaccgctatccacgcccattggtgtactgccaaaaccgc
atcaccatggtaatagcgatgactaatacgtagatgtactgccaagtaggaaagtcccgtaaggtcatgtactgggcataatgccaggcgggccatttaccgtcattgacgtcaataggggggc
ggacttggcatatgatacacttgatgtactgccaagtgggcagtttaccgtaaatactccacccattgacgtcaatggaaagtccctattggcgttactatgggaacatacgtcattattgacgtc
aatgggcgggggtcgttgggcggtcagccaggcgggccatttaccgtaagttatgtaacgcggaactccatatatgggctatgaactaatgaccccgtaattgattactattaataactatcga
gtgtggctaatggagaattgtcgtgccagttgtatcagcgaagcggagatgtcgggttgggcgtgcccttcaatattgcatcatactctcttctgactcatctgatggccagtatggtgggtctaa
aaccgggagagtttatcctcactcttggtgacgcacacatttataatacccacattgaggtgttaaagaagcagttgtgccgcgtccctagaccattccctaagttgaggattttaatggctccag
aaaaaattgaggactttactatcgacatgttttatcttgaggggtatcaaccacacagtggaaacttgcagatgaaaatggctgtttgaatcatgttaaggaatttccttgttactcatttattcctaga
aatggtgtaatcgctgttgtgggcggagcatatttgtgtatataagagcccgtgttagctcctcgattcagtcacaagagcgcacacacacgcttataactagctctctctccactcaaggaattc
aaaggcctacgtcgacgagctcactagtcgcggccgctttcgaatctagagcctgcagtctcgacaagcttgtcgagaagtactagaggatcataatcagccataccacatttgtagaggtttt
acttgctttaaaaaacctcccacacctccccctgaacctgaaacataaaatgaatgcaattgttgttgttaacttgtttattgcagcttataatggttacaaataaagcaatagcatcacaaatttcac
aaataaagcatttttttcactgcattctagttgtggtttgtccaaactcatcaatgtatcttatcatgtctggatctgatcactgcttgagcctaggagatccgaaccagataagtgaaatctagttcca
aactattttgtcatttttaattttcgtattagcttacgacgctacacccagttcccatctattttgtcactcttccctaaataatccttaaaaactccatttccacccctcccagttcccaactattttgtccg
cccacagcggggcatttttcttcctgttatgtttttaatcaaacatcctgccaactccatgtgacaaaccgtcatcttcggctacttt

Supplementary Figure 3

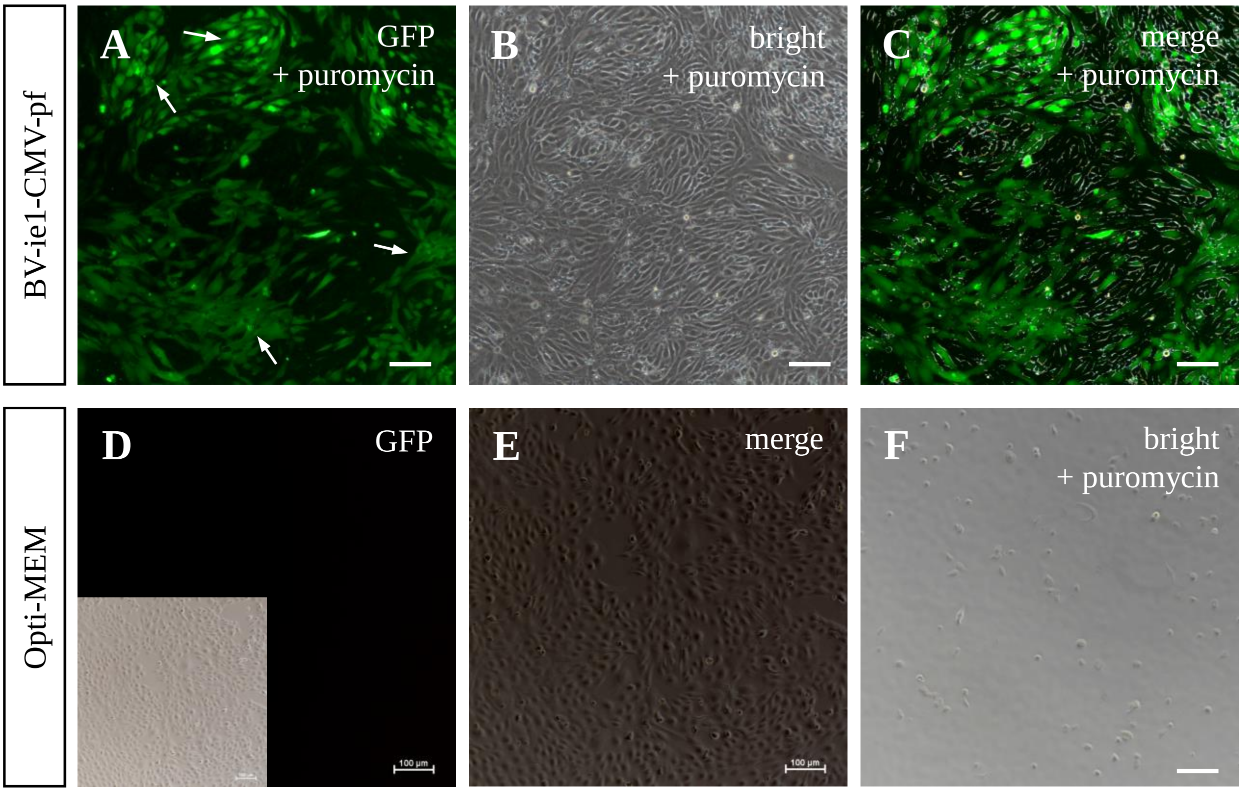

Supplementary Figure 3. ZF4 cells transduced with modified baculovirus BV-ie1-CMV-pf. (**A-C**), ZF4 cell transducted with BV-ie1-CMV-pf where a puromycin-egfp (*pf*) cassette was driven by a CMV promoter at a MOI of 32.5, and cells were treated with 1 μg/mL puromycin continuously for 3 days after 2 days post-transduction. Cells grown quite well under the selection pressure, and arrows indicated that GFP positive cells were in clusters and becoming stable. (**D**, **E**), ZF4 cells treated with opit-MEM medium, (**F**), and selected with 1 μg/mL puromycin for 2 days. The expression of GFP was observed under an inverted fluorescence microscope. Scale bar = 100 μm.

Supplementary Table. Primers used in this study.

Gene/Cassette	Accession number.	Forward primer/Reverse primer (5'-3')		Tm	Size (bp)
WSSV ie1 promoter	AF369029.2	PIE1F	gctgctagcaccatgg**ctcgag**TGTGGCTAATGGAGAATTG	52	501
		PIE1R	tcgacgtaggcctttt**gaattc**CTTGAGTGGAGAGAGAGC	53	
CMV promoter 1		PMF	agcaccatgg**ctcgag**GATCTGACGGTTCACTAAACGA	57	621
		PMR	taatcggtgtgagctGATCAATAATTATCATTAGTTAATGCCC	54	
CMV-pf		PPF	ctcccccatctcccgCTTTACTTGTACAGCTCGTC	53	2046
		PPR	taatcggtgtgagctGATCAATAATTATCATTAGTTAATGCCC	54	
oct4	KY994571	Oct4F	ccgtcagatcctcgaATGACGGAGAGACCGCAGAGC	63	1468
		Oct4R	gtcaccgcatgttagaagacttcctctgccctcGCTGGTGAGGTGACCCACCA	64	
sox2	KY994572	Sox2F	ctaacatgcggtgacgtggaggagaatcccggccctATGTACAACATGATGGAAACCG	56	1001
		Sox2R	ctcccccatctcccgTTACATATGCGATAAGGGAATCGT	57	
klf4	KY994573	Klf4F	gctaccggactcagATGAGGCAGCCACCAAGTGAG	62	1322
		Klf4R	agctgtggtgaattcagggccgggattctcctccacgtcaccgcatgttagaagacttcctctgccctcTAGGTGCCGTTTCATGTGC	58	
CMV promoter 2		CMVF	agtacatcaagtgtatcata**gcggccgc**TAGTTATTAATAGTAATCAATT	60	638
		CMVR	ctcacttggtggctgcctcatCTGAGTCCGGTAGC	58	
oct4-detect	KY994571	Oct4-F	ACTGTACTTCGGCACATTCGT	59	400
		Oct4-R	GCGCTCGGAAACTTGTATGGC	61	
sox2-detect	KY994572	Sox2-F	TCATGCCGTAGCCTCCGTTG	62	385
		Sox2-R	TCAAGAGACCCATGAACGCCTT	61	
klf4-detect	KY994573	Klf4-F	CCTGCGTATCTTCAGTCCAC	57	399
		Klf4-R	GCGGAAAGCCAGAGTAACCC	60	
myc-detect	KY994574	Myc-F	ACGACTACGATTCCTACCAGC	58	312
		Myc-R	ACCCGCTCCACATACAGTCC	61	
β-actin	NM_001104808	Actin-F	CATGTACCCTGGCATTGCT	57	382
		Actin-R	CTCCCCTGTTAGACAACTACCTC	58	
pr		Pr-F	TGAACGGCCACGAGTTCGAGA	63	221
		Pr-R	GCGCTCCCACTTGAAGCCCTC	65	

Capital letters indicated the oligonucleotides specific binding to the templates. The underscores were the sites for restriction digest.
